# The trends of lung cancer burden in BRICS from 1990 to 2021 and its projection to 2035

**DOI:** 10.3389/fonc.2024.1511530

**Published:** 2025-01-03

**Authors:** Yifan Wang, Jingwen Zhu, Shaoqiang Wang, Jihong Zhou

**Affiliations:** ^1^ The Seventh Clinical College of Guangzhou University of Chinese Medicine, Shenzhen, China; ^2^ The Fourth Clinical College of Guangzhou University of Chinese Medicine, Shenzhen, China; ^3^ The Information and Control Engineering College of Qingdao University of Technology, Qingdao, China

**Keywords:** lung cancer, GBD database, burden of disease, risk factors, BRICS

## Abstract

**Background:**

Lung cancer has become the malignant tumor with the highest morbidity and mortality in the world. This study aims to analyze the burden of lung cancer and risk factors associated with lung cancer in the BRICS from 1990-2021 and to project the burden of lung cancer in the BRICS from 2021-2035.

**Methods:**

The Global Burden of Disease (GBD) 2021 database was searched to collect the incidence, prevalence, mortality, disability-adjusted life years (DALYs), and risk factors of lung cancer in the BRICS. Trends in lung cancer burden from 1990-2021 were analyzed using Joinpoint 4.9.1.0, and Bayesian age-period-cohort (BAPC) analyses were performed using R4.4.1 to project the disease burden of lung cancer from 2021-2035.

**Results:**

AAPC(average annual percentage change) and EAPC(estimated average percentage change) of ASIR(age-standardized incidence), ASPR(age-standardized prevalence), ASMR(age-standardized mortality), and ASDR(age-standardized disability-adjusted life year) for lung cancer in Brazil, Russia, and Ethiopia 1990-2021 were less than 0. Egypt’s AAPC and EAPC for ASIR, ASPR, ASMR, and ASDR were all greater than 0 for 1990-2021, and China’s ASIR, ASPR, ASMR, and ASDR were all at the top of the BRICS in 2021. According to BAPC projection Brazil, Iran, Russia, and South Africa will have a decreasing trend in ASIR, ASPR, ASMR, and ASDR from 2021-2035. Egypt will have an increasing trend in ASIR, ASPR, ASMR, and ASDR from 2021-2035. With the exception of Ethiopia, the top tier level 1 and level 2 risk factors in the rest of the BRICS were behavioral factors and smoking factors, respectively.

**Conclusion:**

The BRICS still have a heavy burden of lung cancer, and there are significant differences in the burden of lung cancer among the BRICS. At the same time, many BRICS lung cancer prevention and control measures are worth learning from other developing countries.

## 1 Introduction

Lung cancer, also known as primary bronchopulmonary cancer, refers to malignant tumors originating from the bronchial mucosa or glands that originate in the lungs (1). According to the global cancer statistics report released by the World Health Organization (WHO), in 2022, there were 2.5 million new cases of lung cancer globally, accounting for about one-eighth of all new malignant tumors, and 1.82 million deaths, accounting for about one-fifth of all malignant tumor deaths. Lung cancer has become the malignant tumor with the highest global morbidity and mortality and is the leading cause of cancer morbidity and mortality in men and the 2nd leading cause of cancer morbidity and mortality in females worldwide (1).

BRICS is an international cooperation organization comprising emerging markets and developing countries that aims to promote economic, political, and social cooperation among member countries and to advance the reform of the global governance system. BRICS initially consisted of five countries, Brazil, Russia, India, China, and South Africa. On January 1, 2024, with Saudi Arabia, Egypt, the United Arab Emirates, Iran, and Ethiopia becoming full members of BRICS, the number of BRICS member countries increased from five to ten, and BRICS achieved a historic expansion. Data show that after the expansion of the BRICS accounted for nearly half of the world’s population, nearly 30% of the world’s total economic output, and nearly 40% of the world’s total industrial value (2). The BRICS are facing many problems such as population aging, environmental pollution, imbalance of health resources, high smoking rates, and industrial pollution along with rapid economic and social development, which may lead to an increase in the incidence, prevalence, mortality, and DALYs of lung cancer. Therefore, an in-depth understanding of the burden of lung cancer and the main risk factors behind it in the BRICS, which can be used to optimize public health policies, rationalize the allocation of health resources, and evaluate the effectiveness of interventions, is of great significance in reducing the burden of lung cancer and improving public health.

GBD database covers the incidence rate, morbidity, mortality, and DALYs of lung cancer (3). Therefore, this study quantifies the overall burden of lung cancer in the BRICS based on the GBD database, provides focus and direction for lung cancer prevention and treatment in the relevant countries, and provides data support for health policy decision-makers to accurately and efficiently allocate healthcare resources, identify high-risk populations, and formulate prevention strategies.

## 2 Materials and methods

### 2.1 Data sources

The data of this study were based on the GBD 2021 database published by the Institute for Health Metrics and Evaluation at the University of Washington in the United States of America (4).

### 2.2 Research method

In this study, “tracheal, bronchial and lung cancers” (International Classification of Diseases (ICD) 10 codes: C33-C34.9, D02.1-D02.3, D14.2-D14.3, and D38.1) were selected as the classification criteria for lung cancer. ASIR, ASPR, ASMR, ASDR, and 95% uncertainty intervals (95% UI) for the BRICS(Brazil, Russia, India, China, South Africa, Saudi Arabia, Egypt, UAE, Iran, and Ethiopia) for the years 1990-2021 were extracted from the GBD database (5). And calculate the AAPC, EAPC, and 95% UI of the ASIR, ASPR, ASMR, and ASDR of the BRICS from 1990 to 2021. Simultaneously using BAPC to predict the ASIR, ASPR, ASMR, and ASDR of lung cancer from 2021 to 2035. The GBD2021 database categorizes disease risk factors at four levels, with level 1 covering three major categories of risk factors, including environmental/occupational risks, behavior risks, and metabolic risks, and the subsequent levels continue to be subdivided on this basis (6). In this study, we calculated the proportion of lung cancer deaths attributable to the corresponding risk factors to the total number of lung cancer deaths, the proportion of lung cancer DALYs attributable to the corresponding risk factors to the total lung cancer DALYs, and the change of both from 1990 to 2021, respectively.

### 2.3 Statistical methods

Use Excel 2023 and the tidyverse and reshape2 packages in R 4.4.1 for data organization. The joinpoint regression model is a collection of linear statistical models that were used to evaluate the trends in disease burdens attributable to lung cancer across time. This model’s calculating approach is to estimate the changing rule of illness rates using the least square method, avoiding the non-objectivity of typical trend analyses based on linear trends (7). In this study, Joinpoint 4.9.1.0 was used for AAPC analysis to describe the overall trend of disease burden. The EAPC value is calculated by a linear regression model and is widely used in the field of public health. Its role is to summarize the age-standardized rate trend in a specific time interval (8). This study used the GBDR package in R 4.4.1 for EAPC analysis.The BAPC model is based on the age period cohort analysis (APC) model. However, there is a linear relationship between the three factors in the APC model, which makes parameter estimation difficult. Therefore, a Bayesian model is added on the basis of the APC model. Bayesian model can estimate the prior information of unknown parameters and sample information to obtain the posterior distribution and infer the unknown parameters according to the posterior distribution. The model estimates the posterior edge distribution directly using the integrated nested Laplace approximation (INLA) algorithm. Because the expected effects of time adjacency may be similar, the second-order random walk (RW2) model is used to study the influence of age, period, and cohort, and estimate the number of cases, age-specific incidence, and standardized incidence (9). The BAPC package and INLA package in r.4.4.1 were used for BAPC analysis in this study (10).

## 3 Result

### 3.1 Burden of disease

**Figure 1:**
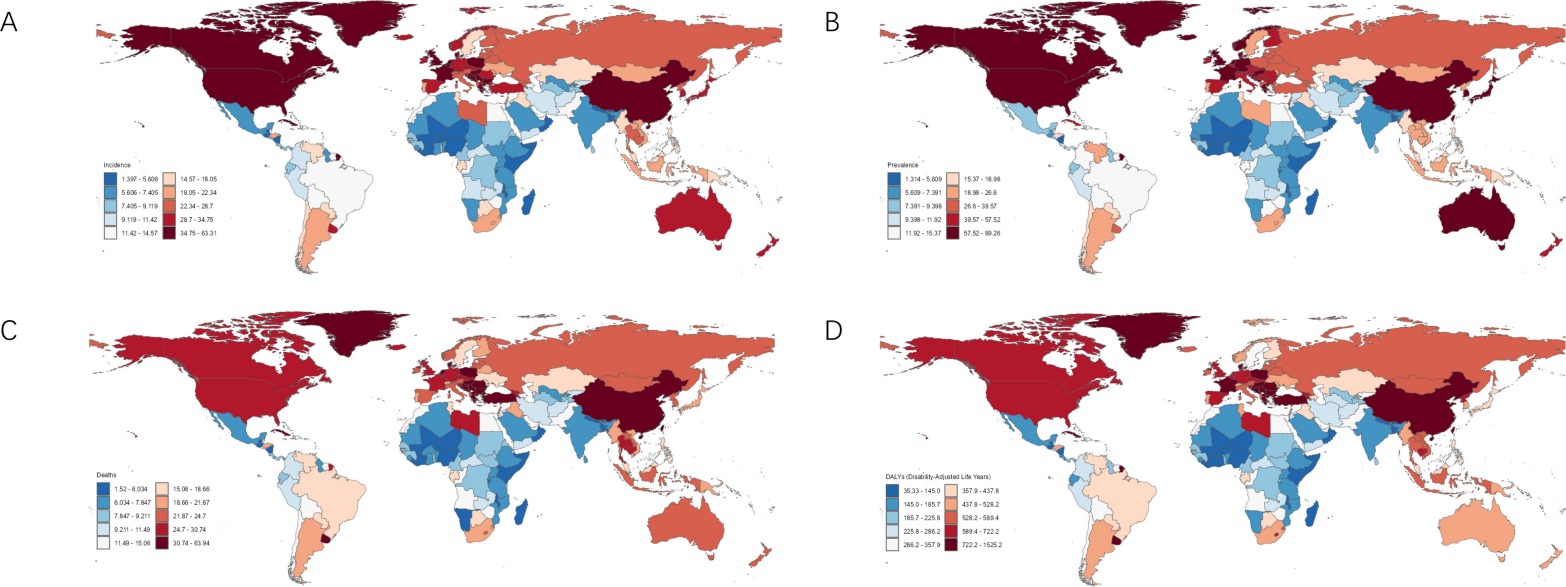
Burden of disease for lung cancer in 204 countries and territories in 2021. **(A)** ASIR; **(B)** ASPR; **(C)** ASMR; **(D)** ASDR.

**Figure 2:**
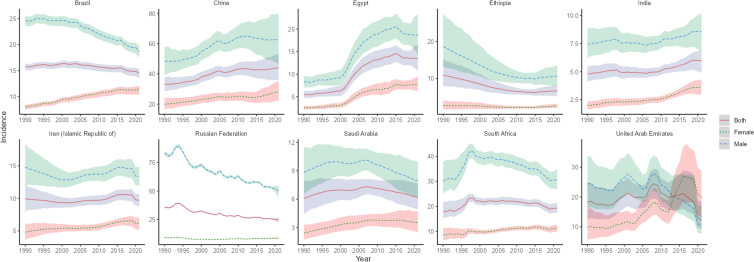
Time trend of ASIR in lung cancer in BRICS from 1990 to 2021. The blue line and its surrounding area represent the projection curves and uncertainty intervals for males; The green line and its surrounding area represent the projection curves and uncertainty intervals for females; The red line and its surrounding area represent the projection curves and uncertainty intervals for both sexes in total.

The country with the highest lung cancer incidence among the BRICS in 2021 was China, with an ASIR of 44.0/100,000 (95% UI: 35.45, 53.35), which ranked 7th out of 204 countries and territories in the world in terms of ASIR, and the lowest was India, with an ASIR of 5.99 per 100,000 (95% UI: 4.92, 6.92), as shown in **Figure 1**. The ASIR for lung cancer in China, Egypt, India, and South Africa all showed an increasing trend (EAPC>0) compared to 1990. The largest increase was in Egypt, with an EAPC of 3.99 (95% CI: 3.42, 4.56), as shown in **Figure 2**, **Table 1**. The ASIR for lung cancer in the rest of the BRICS except China in 2021 was below the global average of 26.43/100,000 (95% UI: 23.90, 29.07).

**Table 1:** ASIR and tends of lung cancer in BRICS.

Country	Age-standardized incidence per 100,000 population (95% UI)		EAPC (95% CI)	AAPC (95% CI)
	1990	2021		
Brazil	15.72(15.05, 16.27)	14.53(13.54, 15.34)	-0.24(-0.32, -0.16)	-0.03(-0.04, -0.03)
China	33.11(28.47, 37.79)	44.01(35.45, 53.35)	1.03(0.88, 1.17)	0.34(0.32, 0.37)
Egypt	5.50(4.72, 6.37)	13.53(10.69, 16.61)	3.99(3.42, 4.56)	0.26(0.25, 0.26)
Ethiopia	10.91(8.07, 15.19)	6.66(5.33, 8.25)	-1.89(-2.15, -1.63)	-0.14(-0.14, -0.14)
India	4.80(4.15, 5.54)	5.99(4.92, 6.92)	0.63(0.47, 0.79)	0.04(0.04, 0.05)
Iran (Islamic Republic of)	9.90(7.98, 12.02)	9.70(8.79, 10.75)	0.24(0.11, 0.37)	-0.01(-0.01, -0.00)
Russian Federation	36.01(35.27, 36.63)	24.69(22.35, 26.86)	-1.35(-1.51, -1.19)	-0.33(-0.37, -0.29)
Saudi Arabia	6.08(4.46, 7.89)	6.19(4.90, 7.69)	0.02(-0.18, 0.22)	0.00(-0.00, 0.01)
South Africa	17.78(15.28, 21.82)	19.24(17.39, 21.47)	0.14(-0.17, 0.45)	0.03(0.00, 0.05)
United Arab Emirates	18.25(12.48, 24.84)	12.43(9.28, 18.37)	-0.04(-0.60, 0.52)	-0.23(-0.31, -0.14)
Gobal	28.54(27.06, 29.91)	26.43(23.90, 29.07)	-0.24(-0.31, -0.17)	-0.07(-0.08, -0.06)

**Figure 3:**
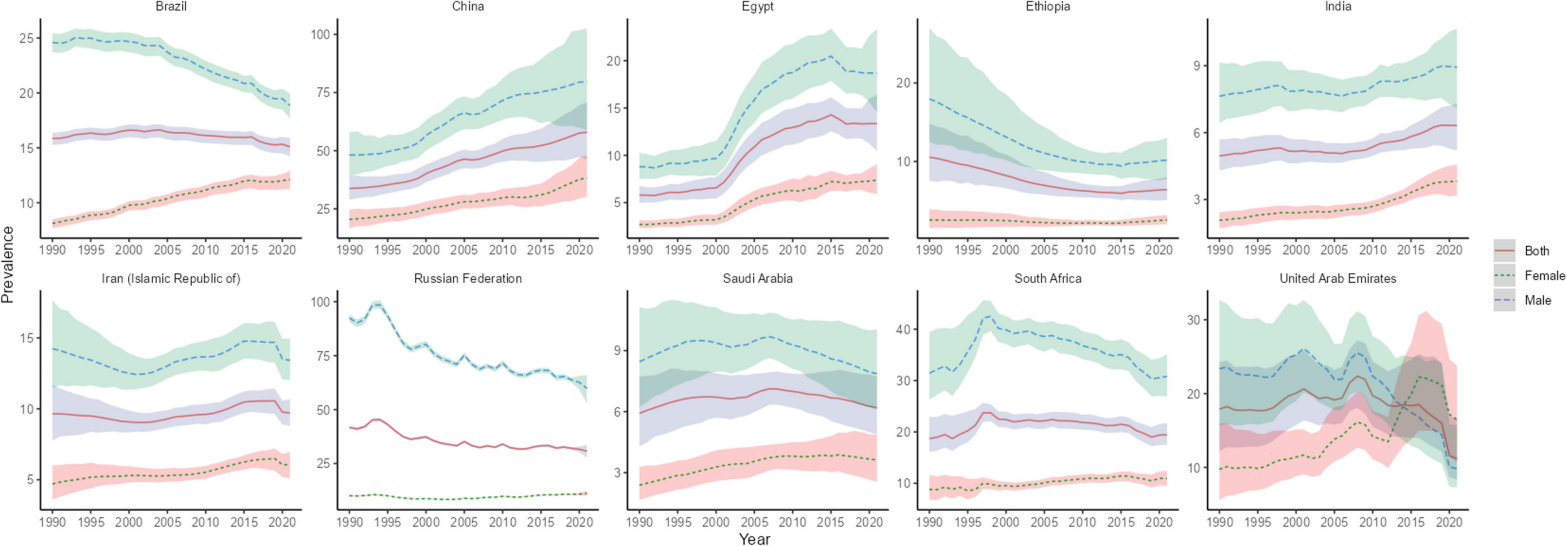
Time trend of ASPR in lung cancer in BRICS from 1990 to 2021. The blue line and its surrounding area represent the projection curves and uncertainty intervals for males; The green line and its surrounding area represent the projection curves and uncertainty intervals for females; The red line and its its surrounding area represent the projection curves and uncertainty intervals for both sexes in total.

The highest lung cancer prevalence in BRICS in 2021 was in China, with an ASPR of 57.95/100,000 (95% UI: 46.20, 70.78), which ranked 18th out of 204 countries and territories in terms of lung cancer ASPR, and the lowest was in India, with an ASPR of 6.32/100,000 (95% UI: 5.19, 7.30), as shown in **Figure 1B**. The ASPR of lung cancer in China, Egypt, India, and Iran showed an increasing trend (EAPC>0) compared with 1990. The largest increase was in Egypt, with an EAPC of 3.68 (95% CI: 3.17, 4.19), as shown in **Figure 3**, **Table 2**. The ASPR for lung cancer in 2021 in all BRICS except China was below the global average of 37.28/100,000 (95% UI: 33.76, 40.77).

**Table 2:** ASPR and trends of lung cancer in BRICS.

Country	Age-standardized prevalence per 100,000 population (95% UI)		EAPC (95% CI)	AAPC (95% CI)
	1990	2021		
Brazil	15.85(15.28, 16.35)	15.09(14.23, 15.89)	-0.16(-0.24, -0.08)	-0.02(-0.03, -0.02)
China	33.74(28.90, 38.65)	57.95(46.20, 70.78)	1.91(1.80, 2.02)	0.77(0.74, 0.80)
Egypt	5.80(4.99, 6.74)	13.35(10.44, 16.42)	3.68(3.17, 4.19)	0.24(0.23, 0.25)
Ethiopia	10.55(7.56, 14.85)	6.41(5.10, 8.02)	-1.93(-2.19, -1.66)	-0.13(-0.14, -0.13)
India	4.96(4.31, 5.71)	6.32(5.19, 7.30)	0.71(0.55, 0.87)	0.04(0.04, 0.05)
Iran (Islamic Republic of)	9.65(7.77, 11.69)	9.71(8.82, 10.68)	0.33(0.18, 0.48)	-0.00(-0.01, 0.00)
Russian Federation	41.76(40.94, 42.43)	30.78(27.76, 33.62)	-1.06(-1.27, -0.85)	-0.32(-0.41, -0.23)
Saudi Arabia	5.92(4.29, 7.73)	6.19(4.86, 7.73)	0.13(-0.05, 0.31)	0.01(0.00, 0.01)
South Africa	18.71(16.07, 22.90)	19.46(17.50, 21.74)	0.00(-0.27, 0.27)	-0.00(-0.03, 0.03)
United Arab Emirates	17.89(12.21, 24.27)	11.16(8.34, 16.53)	-0.56(-1.09, -0.04)	-0.21(-0.26, -0.17)
Gobal	34.25(32.66, 35.69)	37.28(33.76, 40.77)	0.40(0.27, 0.53)	0.09(0.08, 0.10)

**Figure 4:**
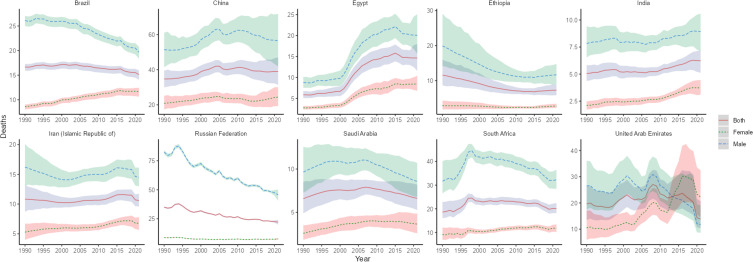
Time trend of ASMR in lung cancer in BRICS from 1990 to 2021. The blue line and its surrounding area represent the projection curves and confidence intervals for males; The green line and its surrounding area represent the projection curves and uncertainty intervals for females; The red line and its surrounding area represent the projection curves and uncertainty intervals for both sexes in total.

The highest lung cancer mortality in BRICS in 2021 was in China with an ASMR of 38.98/100,000 (95% UI: 31.40, 47.06), which ranked 9th out of 204 countries and territories, and the lowest was in India with an ASMR of 6.23/100,000 (95% UI: 5.12, 7.19), as shown in **Figure 1C**. The ASMR of lung cancer in China, Egypt, India, and Iran showed an increasing trend (EAPC>0) compared with 1990. The largest increase was in Egypt, with an EAPC of 4.09 (95% CI: 3.51, 4.68), as shown in **Figure 4**, **Table 3**. The ASMR for lung cancer in the rest of the BRICS except China in 2021 was below the global average of 23.50/100,000 (95% UI: 21.22, 25.85).

**Table 3:** ASMR and trends in lung cancer in BRICS.

Country	Age-standardized rate (per 100000)		EAPC (95% CI)	AAPC (95% CI)
	1990	2021		
Brazil	16.66(15.91, 17.26)	15.12(14.04, 15.98)	-0.28(-0.36, -0.21)	-0.05(-0.06, -0.04)
China	34.74(29.96, 39.52)	38.98(31.40, 47.06)	0.42(0.24, 0.60)	0.15(0.11, 0.18)
Egypt	5.83(4.99, 6.74)	14.63(11.55, 17.96)	4.09(3.51, 4.68)	0.28(0.27, 0.29)
Ethiopia	11.59(8.63, 15.98)	7.27(5.85, 8.98)	-1.79(-2.04, -1.54)	-0.14(-0.14, -0.14)
India	5.05(4.37, 5.83)	6.23(5.12, 7.19)	0.59(0.44, 0.75)	0.04(0.04, 0.05)
Iran (Islamic Republic of)	10.83(8.71, 13.16)	10.61(9.59, 11.76)	0.23(0.10, 0.36)	-0.01(-0.01, -0.00)
Russian Federation	35.00(34.29, 35.64)	22.14(20.09, 24.09)	-1.66(-1.79, -1.52)	-0.39(-0.47, -0.30)
Saudi Arabia	6.58(4.88, 8.54)	6.62(5.26, 8.19)	-0.03(-0.24, 0.18)	0.00(-0.00, 0.00)
South Africa	18.63(16.04, 22.89)	20.14(18.17, 22.44)	0.14(-0.18, 0.45)	0.02(-0.00, 0.05)
United Arab Emirates	19.65(13.51, 26.47)	13.85(10.43, 20.65)	0.15(-0.43, 0.72)	-0.22(-0.32, -0.13)
Gobal	27.58(26.09, 28.99)	23.50(21.22, 25.85)	-0.54(-0.60, -0.47)	-0.14(-0.15, -0.13)

**Figure 5:**
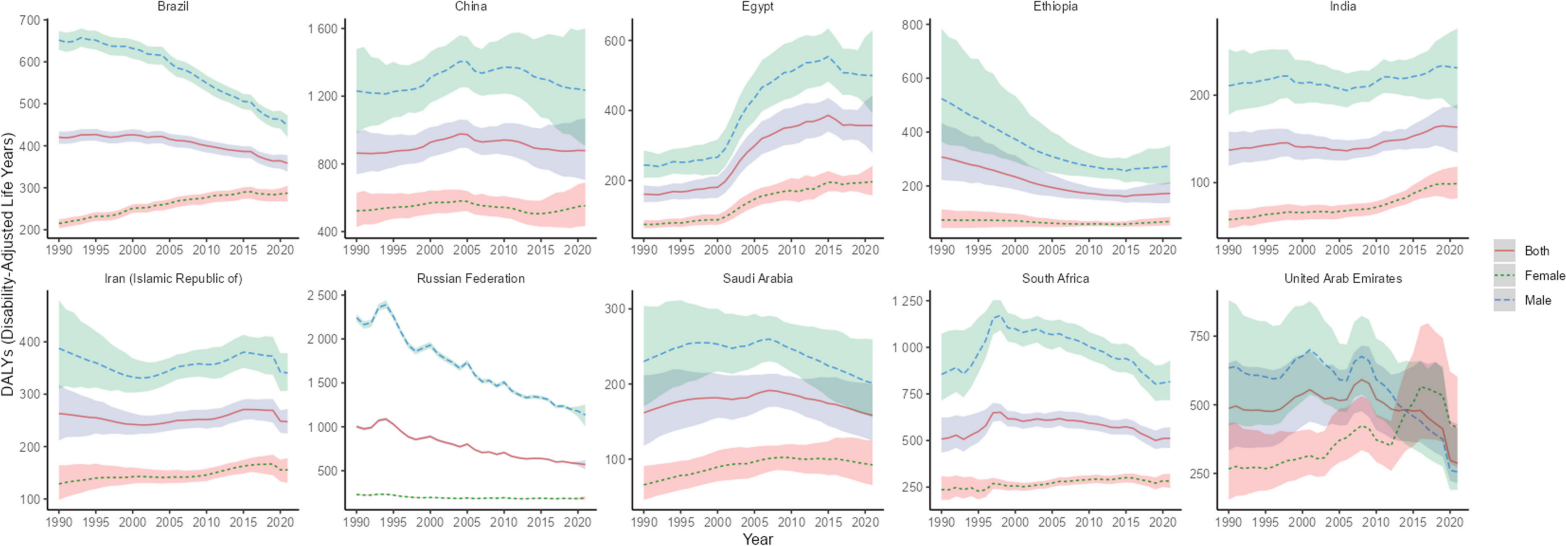
Time trend of ASDR in lung cancer in BRICS from 1990 to 2021. The blue line and its surrounding area represent the projection curves and confidence intervals for males; The green line and its surrounding area represent the projection curves and uncertainty intervals for females; The red line and its surrounding area represent the projection curves and uncertainty intervals for both sexes in total.

The highest lung cancer DALYs in BRICS in 2021 was in China with an ASDR of 878.24/100,000 (95% UI: 703.53, 1068.71), which ranked 12th out of 204 countries and territories, and the lowest was in Saudi Arabia with an ASDR of 158.31/100,000 (95% UI: 124.44, 199.60), as shown in **Figure 1D**. Compared with 1990, Egypt and India ASDR showed an increasing trend (EAPC>0). The largest increase was in Egypt with an EAPC of 6.22 (95% UI:5.98, 6.46), as shown in **Figure 5**, **Table 4**. In 2021 The ASMR for lung cancer in the rest of the BRICS except China in 2021 was below the global average of 533.00/100,000 (480.13, 586.36).

**Table 4:** ASDR and trends in lung cancer in BRICS.

Country	Age-standardized DALYs per 100,000 population (95% UI)		EAPC (95% CI)	AAPC (95% CI)
	1990	2021		
Brazil	420.31(405.09, 434.13)	358.46(338.55, 377.47)	-0.52(-0.61, -0.43)	-1.99(-2.16, -1.83)
China	863.54(738.86, 991.39)	878.24(703.53, 1068.71)	0.07(-0.09, 0.22)	0.66(0.04, 1.29)
Egypt	161.37(138.44, 187.31)	357.30(278.46, 443.28)	3.54(3.03, 4.06)	6.22(5.98, 6.46)
Ethiopia	307.74(223.09, 433.51)	172.44(137.47, 215.52)	-2.20(-2.47, -1.94)	-4.37(-4.45, -4.30)
India	137.23(119.37, 158.43)	163.58(134.43, 189.13)	0.49(0.34, 0.64)	0.85(0.76, 0.94)
Iran (Islamic Republic of)	263.05(211.58, 318.24)	247.42(224.91, 271.78)	0.12(-0.02, 0.26)	-0.60(-0.75, -0.45)
Russian Federation	1003.29(984.26, 1020.47)	569.75(513.85, 621.48)	-2.06(-2.21, -1.90)	-12.88(-14.10, -11.66)
Saudi Arabia	161.60(117.77, 211.66)	158.31(124.44, 199.60)	-0.07(-0.28, 0.13)	-0.12(-0.24, -0.00)
South Africa	507.87(434.31, 623.40)	512.22(459.74, 571.46)	-0.10(-0.41, 0.21)	0.35(-0.63, 1.34)
United Arab Emirates	487.00(333.49, 652.53)	287.29(214.77, 429.88)	-0.75(-1.29, -0.21)	-6.93(-8.69, -5.17)
Gobal	690.86(654.39, 725.97)	533.00(480.13, 586.36)	-0.87(-0.93, -0.81)	-5.20(-5.34, -5.06)

In terms of gender, ASIR, ASPR, ASMR, and ASDR for lung cancer were higher in males than females in all BRICS except UAE, where ASIR, ASPR, ASMR, and ASDR for lung cancer were higher in female than male lung cancer patients after 2014.

### 3.2 Risk factors

The different levels of lung cancer risk factors obtained in this study are as follows: Level 1 includes metabolic, environmental/occupational, and behavioral risks; level 2 includes tobacco (cigarette smoking, secondhand smoke), dietary risks (low-fruit diet), air pollution (ambient particulate matter pollution, indoor air pollution due to solid fuels), other environmental risks (indoor radon contamination), occupational risks (occupational exposure to asbestos, chromium, arsenic, beryllium, nickel, cadmium, diesel engine exhaust, polycyclic aromatic hydrocarbons, and silica), and high fasting blood glucose levels.

**Figure 6:**
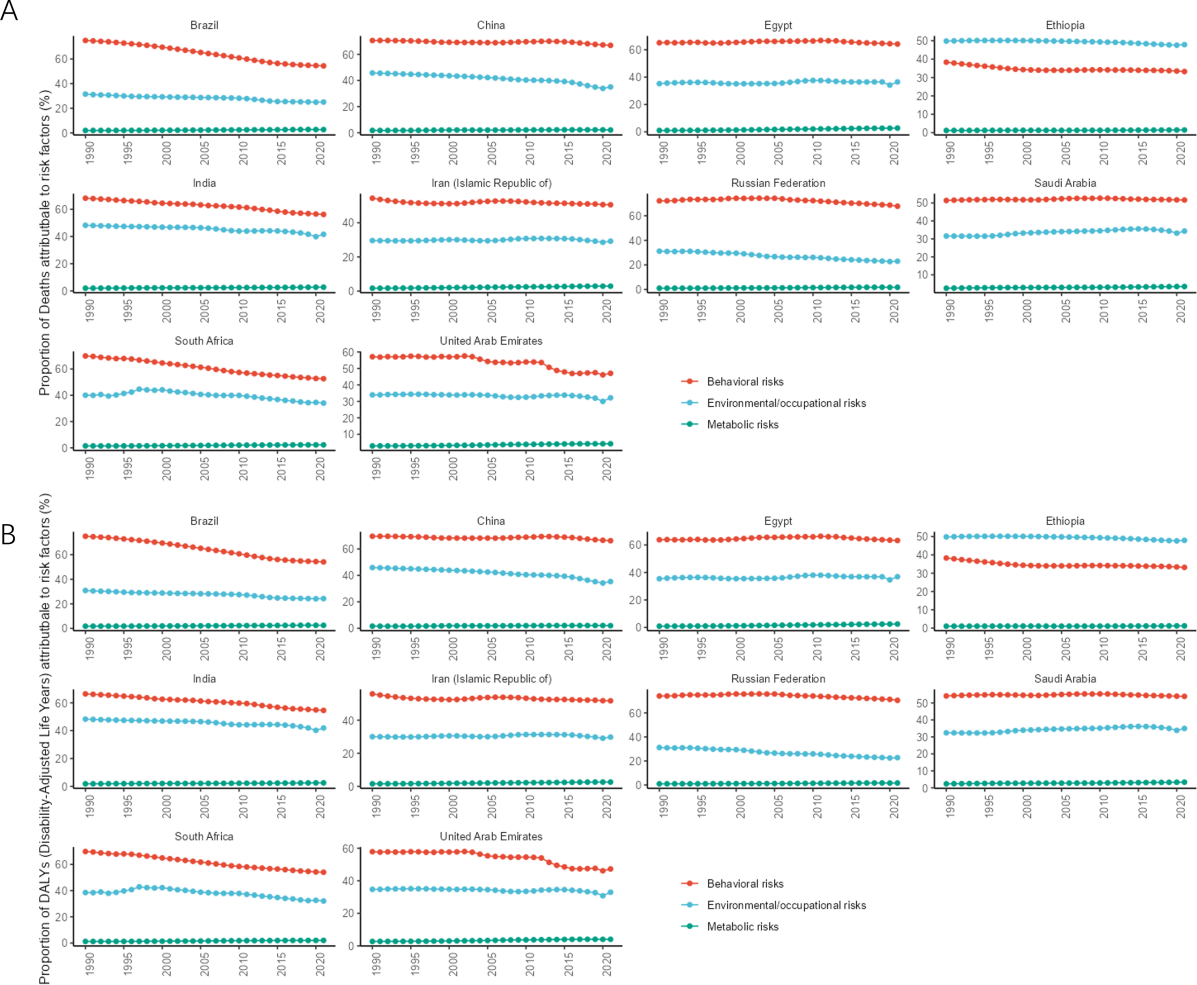
Time trend of the proportion of level 1 risk factors for lung cancer in BRICS from 1990 to 2021. **(A)** Proportion of deaths; **(B)** Proportion of DALYs.

In 2021, among the level 1 risk factors, the ratio of lung cancer deaths attributable to behavioral risks to all lung cancer deaths was highest in all BRICS except Ethiopia, and the ratio of DALYs attributable to behavioral risks to total DALYs was highest in all BRICS except Ethiopia. In 2021, the number of lung cancer deaths attributed to behavioral risks in Russia accounted for 67.77% (95% CI: 63.46, 71.94) of all lung cancer deaths in the country, making it the country with the highest proportion of behavioral risks among BRICS. In 2021, the number of lung cancer deaths attributed to environmental/occupational risks in Ethiopia accounted for 47.88% (95% CI: 35.06, 59.27) of all lung cancer deaths in the country, making it the country with the highest proportion of environmental/occupational risks among BRICS, as shown in **Figure 6A**. DALYs attributable to behavioral risks accounted for 70.47% (95% CI:66.39, 74.63) of total DALYs in 2021 in Russia, the highest percentage of behavioral risks among BRICS. DALYs attributable to environmental/occupational risks accounted for 47.25% (95% CI: 38.92, 55.27) of total DALYs in Ethiopia in 2021, the highest proportion of environmental/occupational risks among BRICS, as shown in **Figure 6B**.

**Figure 7:**
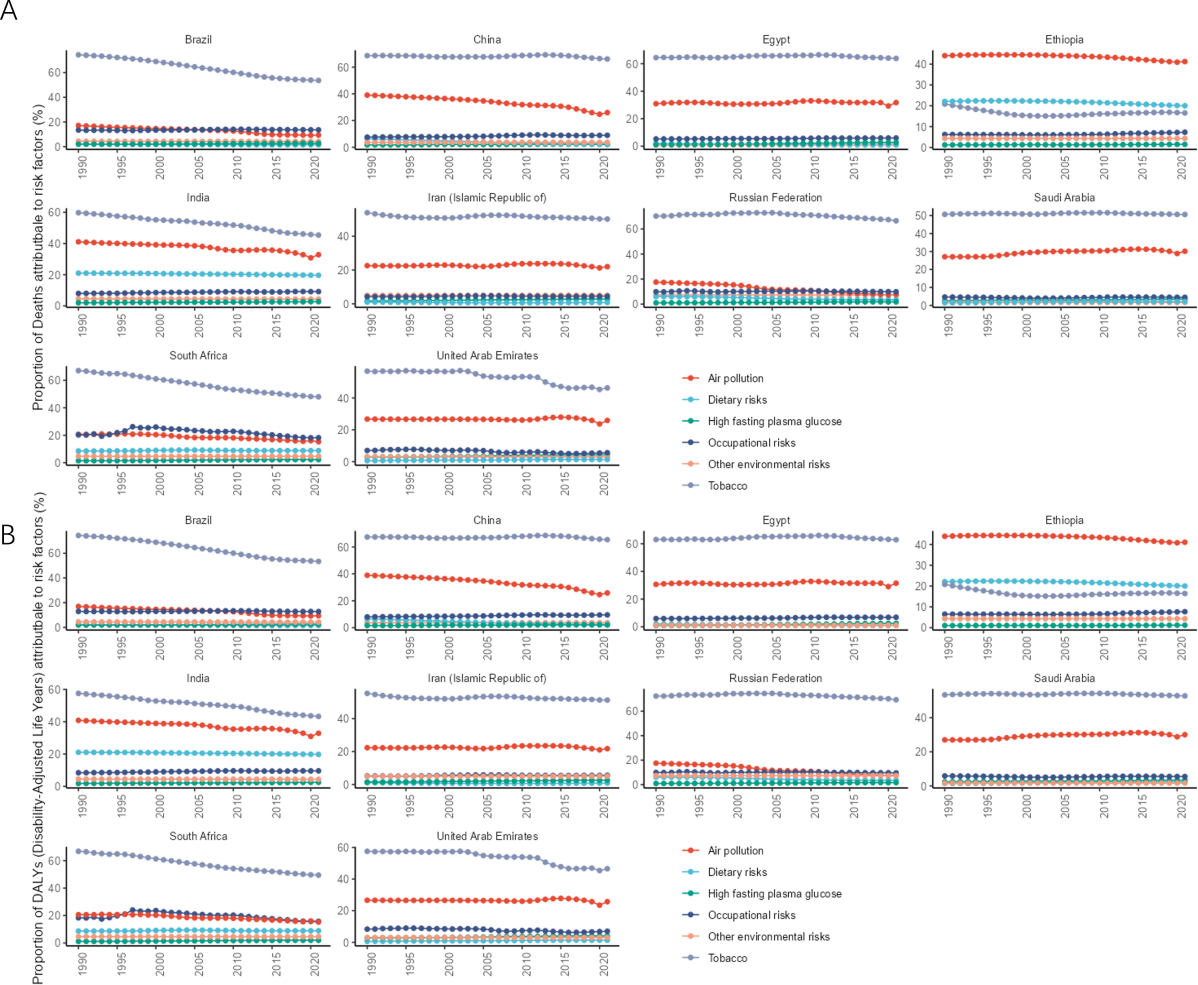
Time trend of the proportion of level 1 risk factors for lung cancer in BRICS from 1990 to 2021. **(A)** Proportion of deaths; **(B)** Proportions of DALYs.

In 2021, among the level 2 risk factors, all BRICS except Ethiopia had the highest proportion of lung cancer deaths attributable to the risk of smoking as a percentage of all lung cancer deaths, and all BRICS except Ethiopia had the highest proportion of DALYs attributable to the risk of smoking as a percentage of total DALYs. The number of lung cancer deaths attributable to the risk of smoking accounted for 66.54% (95% CI: 62.20, 70.97) of all lung cancer deaths in Russia in 2021, making it the BRICS with the highest proportion of smoking risk. In 2021, the number of lung cancer deaths attributed to air pollution in Ethiopia accounted for 41.23% (95% CI: 28.30, 52.80) of all lung cancer deaths, making it the country with the highest proportion of air pollution risk among BRICS, as shown in **Figure 7A**. In 2021, DALYs attributed to smoking risks accounted for 69.34% (95% CI: 65.11, 73.55) of the total DALYs in Russia, making it the country with the highest proportion of smoking risks among BRICS. In 2021, DALYs attributed to air pollution accounted for 41.06% (95% CI: 28.16, 52.61) of the total DALYs in Ethiopia, making it the country with the highest proportion of air pollution risks among BRICS, as shown in **Figure 7B**.

### 3.3 Projection of disease burden trends

**Figure 8:**
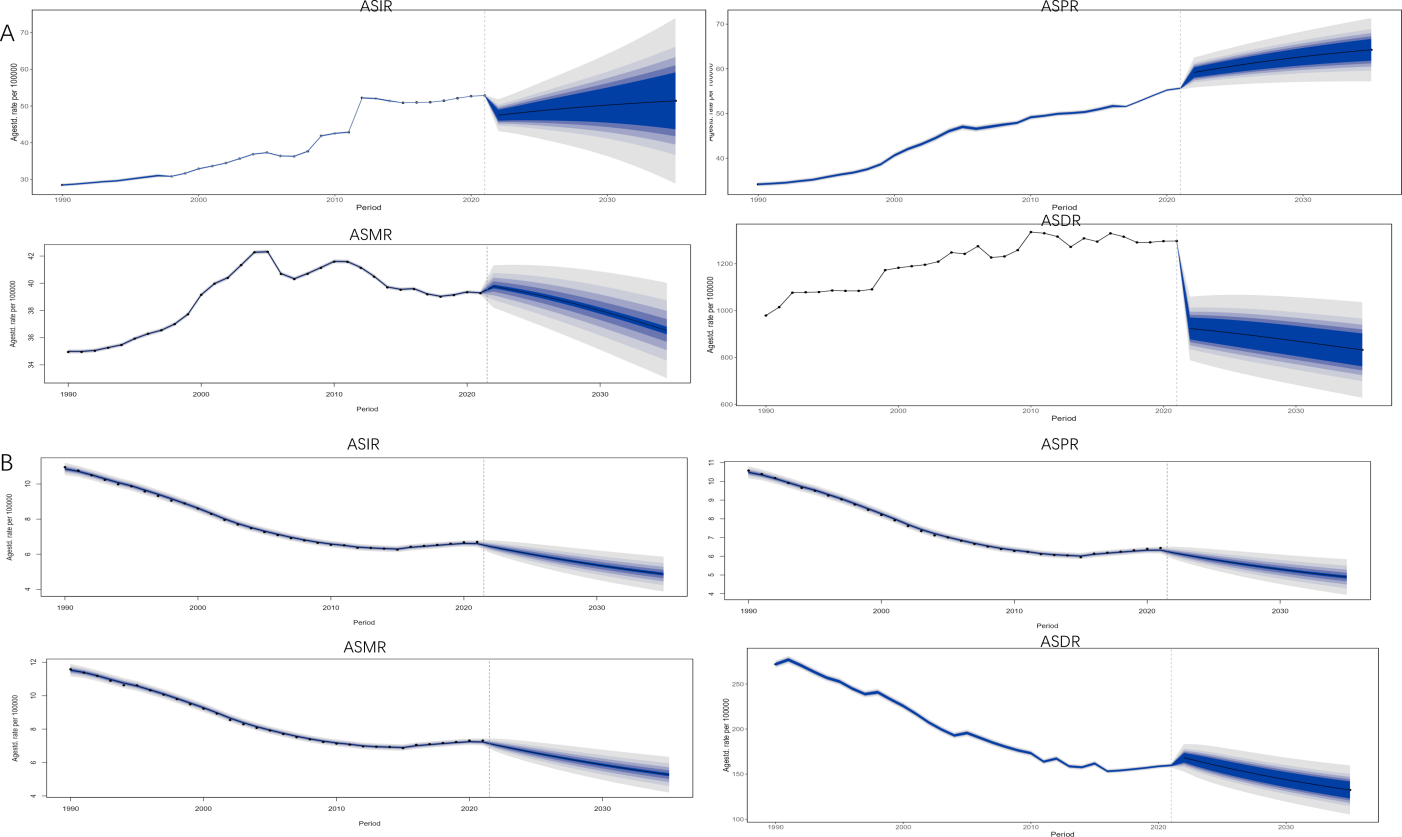
Projection of lung cancer ASIR, ASMR, ASMR, and ASDR trends in BRICS from 2021 to 2035. The blue area represents 95% UI. **(A)** China; **(B)** Ethiopia.

According to BAPC projection Brazil, Iran, Russia, and South Africa will have a decreasing trend in ASIR, ASPR, ASMR, and ASDR from 2021-2035. Egypt will have an increasing trend in ASIR, ASPR, ASMR, and ASDR from 2021-2035. By 2035, China’s lung cancer ASIR [50.96/100,000 (95% UI:28.90, 73.02)], ASPR [63.61/100,000 (95% UI:56.55, 70.68)], ASMR [36.53/100,000 (95% UI:32.38, 40.69)] and ASDR [916.28/100,000 (95% UI:500.19, 1332.37)] will be the highest in the BRICS. China’s lung cancer ASIR, ASPR, and ASDR will be in an upward trend during 2021-2035, but China’s lung cancer ASMR will be in an upward trend during 2021-2032 and then in a downward trend during 2022-2035, as shown in **Figure 8A**. By 2035, Ethiopia’s lung cancer ASIR [4.87/100000 (95% UI: 3.70, 6.04)], ASPR [4.89/100000 (95% UI: 3.77, 6.01)], ASMR [5.27/100000 (95% UI: 4.02, 6.53)], and ASDR [132.4/100000 (95% UI: 105.13159.66)] will all be the lowest among BRICS, as shown in **Figure 8B**.

## 4 Discussion

As an emerging global multilateral cooperation mechanism and the most rapidly developing economic cooperation organization today, BRICS has gradually become an important force in global governance, but due to factors such as the continuous advancement of population aging, the frequent occurrence of epidemics, the problem of accessibility to medicines, the acceleration of the urbanization process, the changes in lifestyle brought about by economic development, and the relatively weak public health capacity building in developing countries, BRICS are also facing common public health security challenges (11, 12). In 2021, BRICS accounted for approximately 50.16%, 45.69%, 49.72%, and 51.83% of global lung cancer deaths, prevalence, incidence, and DALYs, respectively. There are also large differences in the burden of lung cancer, predicted trends, and risk factors among BRICS.

Brazil is the largest and most populous country in Latin America, and the largest emerging economy in Latin America. The AAPC and EAPC of ASIR, ASPR, ASMR, and ASDR of lung cancer in Brazil were all less than 0 in 1990-2021, and according to the BAPC projection, from 2021 to 2035, ASIR, ASPR, ASMR, and ASDR will all be in a decreasing trend. This may be related to the smoking ban and environmental protection measures in Brazil. Since 1990, the Brazilian government has formulated a number of powerful anti-smoking measures, such as banning smoking in public places, raising the tax rate of tobacco products, and setting health warning labels on cigarette packages (13). In 2006, the smoking population in Brazil accounted for 15,7% of the national population, while by 2018, the smoking population accounted for only 9,3% of the national population, a decrease of nearly 40% (14). In terms of environmental governance, Brazil’s Air Pollution Control Program for Motor Vehicles was established in 1987 to reduce vehicle air pollution. Brazil also established a National Programme for Control of Air Quality in 1989 which established strategies for setting national standards for air quality and emissions at source (15). Although the burden of lung cancer in Brazilian males was in a decreasing trend between 1990 and 2021, the ASIR, ASPR, ASMR, and ASDR of lung cancer in Brazilian females were in an increasing trend between 1990 and 2021. Although there were fewer female smokers compared to males, the reduction in the number of smokers in females was also lower compared to males (16). According to the findings, females are 31% less likely to quit smoking than men, and the percentage of females who succeed in quitting smoking is also lower compared to men (17). Moreover, the type of lung cancer with high incidence in females is lung adenocarcinoma, and the type of lung cancer with high incidence in males is lung squamous cell carcinoma. Compared with lung squamous cell carcinoma, the relationship between lung adenocarcinoma and smoking is relatively low. Therefore, after the decline in the proportion of smokers, the decline in the burden of lung cancer in women is less significant than that in men (18). Based on the above, the Brazilian government should pay more attention to females’ lung cancer burden in the future of lung cancer prevention and control.

China is one of the permanent members of the United Nations Security Council, the third largest country in the world in terms of land area and the second largest economy in the world. In 2022, the number of female lung cancer cases accounted for 17.5% of female malignant tumors in China, surpassing that of breast cancer (15.6%), and in China lung cancer ranked first in terms of morbidity and mortality among malignant tumors for both males and females (19, 20). China’s ASIR, ASPR, ASMR, and ASDR of lung cancer were the first in BRICS, and the AAPC and EAPC of China’s lung cancer ASIR, ASPR, and ASMR were greater than 0 from 1990 to 2021, the EAPC95% UI of ASDR passed through 0, and the AAPC of ASDR was greater than 0. The projection of BAPC showed that from 2021-2035 China’s lung cancer ASIR, ASPR, and ASDR will be in an upward trend. Studies have shown that the prevalence of smoking among Chinese males is as high as 60% (21), and the prevalence of smoking among Chinese females and adolescents is rapidly increasing (22). The willingness of Chinese smokers to quit is low, only 16.1% of smokers plan to or consider quitting (21). Meanwhile, with the rapid development of industrialization and transportation in China, especially the extensive use of coal and petroleum, several hazardous substances have been emitted into the atmosphere, exacerbating the high lung cancer disease burden in China (23). Previous studies have shown that there is a dose-response relationship between lung cancer risk and exposure to cooking fumes and that Chinese cooking is typically more likely to result in the evaporation of fumes compared to its regional cooking methods (24). Not only that, China has one of the highest levels of population aging in the world, but also the highest level of population aging among the BRICS (25), and the increase in the proportion of the older adult population has exacerbated the disease burden of lung cancer. Although China’s economic development and medical level have significantly improved after the reform and opening up, there is still a huge gap in the level of medical security between urban and rural areas. According to data from the National Bureau of Statistics of China, the per capita medical and health expenditure of urban residents in 2023 (2850 yuan) was 1.49 times that of rural residents (1916 yuan). At the same time, in 2023, the per capita financing of employee medical insurance (6182 yuan) was 5.63 times that of urban and rural residents’ medical insurance (1098 yuan). The above data all indicate an extreme imbalance in the allocation of medical resources between urban and rural areas in China, which exacerbates the burden of lung cancer disease on rural areas and farmers in China (26). However, according to predictions, the ASMR of lung cancer in China is expected to decrease in 2032-2035, which may be due to the popularization of clean energy, the improvement of healthcare and education levels, and the decrease in smoking rates among adolescents (27). The gender difference in lung cancer disease burden in China was very obvious, with ASIR, ASPR, ASMR, and DALYs for lung cancer in males being more than twice as high as those in females. The smoking rate of Chinese males is much higher than that of females, which may be one of the reasons for the significant gender differences in lung cancer burden in China (28). Another possible reason is that men and women have different sensitivities to tobacco carcinogens. However, studies have shown that the risk of lung cancer in Chinese male nonsmokers is also higher than that in female nonsmokers, while in Western populations, the risk of lung cancer in male nonsmokers is very low and not higher than that in women (29, 30). To sum up, China’s rapid economic growth has also caused environmental problems. The aggravation of environmental problems has increased the burden of lung cancer. Coordinating the contradiction between economic development and the environment, promoting the upgrading of industrial structure, dealing with the problem of uneven distribution of medical resources, slowing down the aging process of the population, and promoting stricter tobacco control policies are the key and difficult points for the Chinese government’s disease prevention and control in the future.

Egypt is one of the initiators of the Arab League and plays an important role in Arab, African, and international affairs. It is the third largest economy in Africa. The AAPC and EAPC of ASIR, ASPR, ASMR, and ASDR of lung cancer in Egypt were greater than 0 in 1990-2021, and according to the BAPC, it was predicted that ASIR, ASPR, ASMR, and ASDR of lung cancer in Egypt would be in an upward trend in the period of 2021-2035, which may be closely related to the turbulent political situation in Egypt. Egypt faced serious political divisions and social unrest after the Arab Spring, and the political turmoil caused by the Arab Spring led to frequent changes in government disrupted the continuity of health policies, and affected the implementation of public health programs (31). In terms of risk factors, according to WHO statistics, about 22% of the Egyptian population are smokers(without counting hookah), Egypt consumes about 20 billion cigarettes per year, and the prevalence of smoking among Egyptian adolescents is increasing (32). Under the dual influence of politics and lifestyle (smoking), the burden of lung cancer in Egypt is not optimistic. Reducing the smoking rate and maintaining social stability are important ways to reduce the burden of lung cancer in Egypt.

Ethiopia is the fifth largest economy and second largest population country in Africa, and a member of the African Union. The headquarters of the African Union and the United Nations Economic Commission for Africa are both located in Ethiopia. The AAPC and EAPC of ASIR, ASPR, ASMR and ASDR of lung cancer in Ethiopia were less than 0 in 1990-2021, and based on BAPC, it was predicted that ASIR, ASPR, and ASMR of lung cancer in Ethiopia would be in a decreasing trend in the period of 2021-2035 and that the DALYs of lung cancer in Ethiopia will be on the rise in the period of 2021-2022 after which 2022-2035 will be in decreasing trend. Although Ethiopia’s total and per capita GDP is at the bottom of the BRICS, Ethiopia’s GDP maintained a high average annual growth rate of 10% between 2000 and 2010, and between 1990 and 2023, Ethiopia’s total GPP increased 5.12 times, and per capita GDP increased 4.96 times, making it one of the fastest growing economies. Ethiopia’s economic development has led to investment in public health infrastructure and increased capacity for early screening and treatment of lung cancer (33). In terms of risk factors, Ethiopia is the only BRICS country where environmental/occupational risks rank first in level 1 risk factors and air pollution risks rank first in level 2 risk factors. First of all, Ethiopia’s rapid economic development has caused environmental damage (34). Secondly, in Ethiopia, especially in rural areas, due to the lack of clean energy and modern energy facilities, many households rely on biomass fuels (such as wood, crop residues, and animal manure) for cooking and heating. These fuels will produce a large number of harmful air pollutants during combustion, leading to indoor air quality problems. In terms of behavioral risk, Ethiopia’s degree of industrialization and urbanization is lower than that of other BRICS, while the high industrialization and urbanization of other BRICS bring higher living standards and also lead to an increase in behavioral risks such as smoking. Therefore, Ethiopia’s behavioral risk has a lower impact on lung cancer than other BRICS.

India is the largest country in the South Asian subcontinent, the most populous country in the world, and the fifth largest economy in the world. The AAPC and EAPC of ASIR, ASPR, ASMR, and ASDR of lung cancer in India for the period 1990-2021 were greater than 0. Meanwhile, according to BAPC projection, India’s ASIR, ASMR, and ASDR will be in an upward trend in 2021-2022 after declining in 2022-2035 and India’s ASPR will be in an upward trend during 2021-2035. India is the world’s second-largest consumer of tobacco after China, and smoking prevalence among males in India nearly tripled between 1998 and 2010, with a particularly marked increase among younger age groups (35, 36). Rapid economic development, industrialization, and urbanization in India have increased energy consumption and industrial waste emissions, leading to a growing air pollution problem in India. In numerous parts of India, where air pollution levels far exceed the safety standards set by WHO (24-hour ambient PM2.5 standard of 15 μg/m³, not to be exceeded more than 3 to 4 times per year), such as Delhi, the capital of India, has an average PM2.5 of up to 100 µg/m³ in 2021. Burning celebrations during festivals and burning of crop residues in India can even elevate PM2.5 to 700-1000 μg/m³ (37). Although the Indian constitution stipulates that all citizens can enjoy free healthcare, the government’s investment in healthcare is very limited. According to WHO data, in 2021, India’s healthcare expenditure accounted for 3.28% of GDP, which is only slightly higher than Ethiopia’s (3.21%) among BRICS (38). Therefore, under the multiple influences of insufficient medical expenditure, lifestyle (smoking), and environmental issues, the burden of lung cancer disease in India is increasing.

Iran is located in the heart of West Asia, known as the “Eurasian land bridge” and “East-West air corridor”, and is the world’s largest Shiite Islamic country with rich oil and gas resources. ASIR, ASMR, ASPR, and ASDR of lung cancer in Iran 1990-2021 showed large fluctuations, especially during 2019-2021. The EAPC of ASIR, ASMR, and ASPR for lung cancer in Iran between 1990-2021 was greater than 0, and the 95% uncertainty interval of the EAPC of ASDR passed through 0. The EAPC of ASIR, ASMR, and ASPR for lung cancer between 1990-2021 was less than 0, and the 95% uncertainty interval of the AAPC of ASPR passed through 0. According to BACPs projection, ASIR, ASPR, ASMR, and ASDR of lung cancer in Iran will be in a decreasing trend between 2021-2035. The reason for this phenomenon may be due to the fact that Iran was affected by the COVID-19 outbreak leading to the distortion of the data, which in turn led to huge fluctuations in the data related to lung cancer in 2019-2021. In Iran, lung cancer ranks 4th and 5th in the order of incidence and mortality of malignant tumors in males and females, respectively (39). According to the National Cancer Registration Program (NCRP) of Iran, the incidence of lung cancer in Iran increased by an average of 6.8% per year in males and 7.7% per year in females during the period from 2000 to 2016 (39). The increased burden of lung cancer in Iran may be closely related to influences such as high smoking prevalence (39), environmental pollution (40), economic sanctions (41), and gas warfare during the Iran-Iraq war (42).

Russia is one of the permanent members of the United Nations Security Council and the largest country in the world. AAPC and EAPC of ASIR, ASPR, ASMR, and ASDR for Lung Cancer in Russia from 1990 to 2021 were less than 0. According to BACPs projection, lung cancer ASIR, ASPR, ASMR & ASDR in RUS will be on a downward trend between 2021-2035. The reason for the improvement in the burden of lung cancer in Russia may be related to a series of tobacco control measures taken by the Russian government (43), such as the signing of the WHO Framework Convention on Tobacco Control in 2008 and subsequent measures. From 2009 to 2016, the smoking rate in Russia decreased by 21.5% (44). Although the ASIR, ASPR, ASMR, and ASDR of lung cancer in Russia have a downward trend, and the burden of lung cancer in Russian females is low in BRICS, the ASIR, ASPR, ASMR, and ASDR of lung cancer in Russian males are still at a high level. First of all, this may be related to the low smoking rate of Russian females (45). Secondly, industry plays an important role in the Russian economy, especially the energy industry and heavy industry. The work related to it may involve occupational exposure related to lung cancer, such as asbestos, cadmium, and silicon (46). The proportion of males in these jobs is often high. Hence, the burden of lung cancer on males in Russia is still worthy of attention.

Saudi Arabia has the second largest sand oil reserves in the world, is one of the major members of the Organization of Petroleum Exporting Countries (OPEC), has the second largest total GDP in the Middle East, is the world’s largest Sunni Islamist country, and has a pivotal political and economic influence in the Middle East. The 95% uncertainty intervals for the EAPC of ASIR, ASPR, ASMR, and ASDR for lung cancer in Saudi Arabia 1990-2021 passed through 0. The 95% uncertainty intervals for the AAPC of ASIR and ASMR all passed through 0. The AAPC of ASPR was greater than 0, and the AAPC of ASDR was less than 0. According to BACP projection, ASIR for lung cancer in Saudi Arabia will be declining in 2021-2031 and will be in an upward trend in 2031-2035, ASPR will be in an upward trend in 2021-2035, ASMR will be in a downward trend in 2021-2035, ASDR will be in a downward trend in 2021-2023, and in an upward trend in 2024- 2035 will be on an upward trend. It is worth noting that ASIR, ASPR, ASMR, and ASDR of lung cancer in Saudi Arabia were in a continuous downward trend from 2007 to 2021, and in 2021 Saudi Arabia’s ASIR, ASPR, ASMR of lung cancer was only higher than India’s among the BRICS and in 2021 Saudi Arabia’s ASDR was ranked the lowest among the BRICS. This may be related to Saudi Arabia’s strict Islamic doctrine, which banned smoking until the 1960s. Although smoking is now legal, it is still considered a condemned behavior at the religious level in Saudi Arabia. Saudi Arabia passed the WHO Framework Convention on Tobacco Control in 2015 and began imposing heavy taxes on cigarettes after 2017 (47). However, although the Ministry of Commerce and Investment of the Kingdom of Saudi Arabia formally implemented the ban on the sale of electronic cigarettes in September 2015, in August 2019, the Saudi Food and Drug Administration began to implement the technical regulation SFDA FD.60 for tobacco products, which opened the way for the opening and compliance of the electronic cigarette market in Saudi Arabia, and the regulation does not restrict the Internet sales of electronic cigarettes. Therefore, electronic cigarette products in Saudi Arabia are allowed to be sold through online channels at present (48). In addition, with the implementation of Saudi Arabia’s “Vision 2030” policy and the advancement of secularization, Saudi Arabia will still face challenges such as environmental pollution and changes in lifestyle habits.

Located at the southernmost tip of the African continent, South Africa is the second largest economy in Africa and the most economically developed and industrialized country in Africa. The 95% uncertainty intervals for the EAPC of ASIR, ASPR, ASMR, and ASDR for lung cancer from 1990-2021 passed through 0. The 95% uncertainty intervals for the AAPC of ASPR, ASMR, and ASDR passed through 0, and the AAPC of ASIR was greater than 0. According to BACP projection, lung cancer ASIR, ASPR, ASMR, and ASDR in South Africa will be on a declining trend between 2021-2035. South Africa has implemented strict tobacco control policies such as restricting smoking in public places and increasing excise taxes on cigarettes since 1993 (49). The per capita cigarette consumption in South Africa decreased by 54% from 1999 to 2011 (50). However, on the other hand, South Africa has a huge gap between the rich and the poor. According to Statistics South Africa, the Gini coefficient of South Africa is 0.67, which belongs to the high-income inequality countries. At the same time, South Africa’s health care system is divided into the public (83%) and the private (17%) sectors, the public health care system suffers from understaffing and a lack of resources, in contrast to the private system which has sufficient resources staffing and advanced and modern treatments. As a result, early screening and effective treatment of lung cancer are often difficult to access for low-income groups in South Africa (51). In the future, the huge gap between the rich and the poor and the imbalance in the distribution of healthcare resources may be a problem to be faced in the future of lung cancer prevention and control in South Africa. In terms of regulating electronic cigarettes, South Africa proposed the Control of Tobacco Products and Electronic Delivery Systems Bill in 2018, which aims to regulate electronic cigarette products as tobacco products rather than as drugs or consumer goods. However, due to strong opposition from the electronic cigarette industry and lobbying activities, the bill has not yet been officially enacted into law (52). In the future, how to reduce the wealth gap and alleviate the imbalance in the allocation of medical resources, as well as how to strengthen the regulation of electronic cigarette products, will be the problems that South Africa’s lung cancer prevention and control will face.

The United Arab Emirates is located at the southeastern end of the Arabian Peninsula, facing Iran across the sea. It is a maritime transportation hub that controls the Persian Gulf and enters the Indian Ocean, with abundant oil and gas resources. At the same time, the United Arab Emirates is also the country with the highest per capita GDP in the Middle East and an important hub for East-West trade. The AAPC and EAPC of ASIR, ASPR, and ASDR for lung cancer in the UAE 1990-2021 were less than 0, the AAPC of ASMR was less than 0, and the 95% uncertainty interval of the EAPC of ASMR passed through 0. According to BACP projection, lung cancer ASIR, ASPR, and ASMR in the UAE will be in a declining trend between 2021-2035, ASDR will rise in 2021-2022 and be in a declining trend in 2023-2035. It is worth noting that the ASIR, ASPR, ASMR, and ASDR for lung cancer in UAE females exceeded those of males after 2014, and the UAE is the only BRICS country to have a higher lung cancer burden indicator for females than for males. The UAE 2023 census data shows that the gender ratio in the UAE shows a large disparity, with males comprising 68.58% of the country’s population and females comprising 31.42% (53). The oil boom changed the economic landscape of the Gulf region and driven by the oil industry, Gulf countries such as the UAE entered an era of large-scale importation of foreign labor in the 1970s, with the number of expatriates in the UAE exceeding the number of locals. According to 2017 data, the UAE has the highest percentage of expatriate working population among the BRICS and Gulf countries, and the expatriate working population is mainly concentrated in young males (54), with a relatively low incidence of lung cancer, which may have led to an imbalance in the male-to-female ratio in the UAE and a lower burden of lung cancer in males than in females after age standardization after 2014. The other reason may be due to the increasing popularity of smoking behavior with the advancement of secularization in the UAE, especially hookah is becoming more popular among females (55). Meanwhile, studies have shown that higher levels of estrogen in females have a promoting effect on lung cancer (56), making them more sensitive to tobacco carcinogens and more susceptible to lung cancer than males under the same exposure conditions (57). The above reasons may have contributed to the higher indicators of lung cancer burden among females than males in the UAE. Therefore, for the UAE, protecting the medical rights and interests of migrant workers and reducing the female smoking rate will be the focus of lung cancer prevention and control in the future.

There are some limitations in this study, firstly, the fact that all BRICS members are developing countries, which may not be able to provide comprehensive and accurate disease and health data due to resource and infrastructure limitations. Secondly, there are significant regional and urban-rural differences within developing countries represented by China, India, Russia, etc., but the data in the GBD database cannot fully reflect these differences. Moreover, the economic growth and health data of developing countries represented by Iran, Egypt, and Russia are extremely vulnerable to external economic and political factors, which may lead to bias in BAPC prediction. At the same time, the policy adjustments of electronic cigarettes in different countries in the future may also bias the BAPC prediction of diseases. Thirdly, according to the WHO report in August 2020, since the outbreak of COVID-19, 90% of the world’s key medical services have been affected, while cancer diagnosis and treatment have been affected as much as 55% (58). In addition, studies have shown that during the initial peak period of the COVID-19 epidemic, many countries have reported a significant decline in lung cancer cases, which suggests that the epidemic has an important impact on cancer diagnosis, and the mortality of lung cancer patients with COVID-19 is as high as 30%~50% (59). Therefore, the epidemic of COVID-19 may have a certain impact on the statistics of incidence rate and mortality of lung cancer, thus causing data bias.

## 5 Conclusion

In general, although BRICS are all developing countries, due to the influence of living habits, national policies, economic development level, population aging, politics, religion, war, and other factors, there are also great differences in the burden of lung cancer among countries. Understanding these differences can enable BRICS to make more accurate decisions in future public health construction, and to realize the rational allocation of resources. At the same time, although BRICS still have a heavy burden of lung cancer, the strict tobacco control policies of Brazil, Russia, South Africa, and Saudi Arabia, the environmental protection policies and energy structure adjustment policies of China and Brazil, and Ethiopia’s increased investment in medical construction have all played a positive role in reducing the burden of lung cancer. These policies also provide ideas for the prevention and control of lung cancer in other developing countries.

## Data Availability

The original contributions presented in the study are included in the article/supplementary material. Further inquiries can be directed to the corresponding author.

## References

[1] Leong TL Steinfort DP. Contemporary concise review 2023: advances in lung cancer and interventional pulmonology. Respirology. (2024) 29:665–73. doi: 10.1111/resp.14789 38960450

[2] Xin HS. (2024). Available online at: https://www.gov.cn/yaowen/liebiao/202405/content_6949027.htm (Accessed September 21, 2024).

[3] Zhang M Jin W Tian Y Zhu H Zou N Jia Y. Cancer burden variations and convergences in globalization: a comparative study on the tracheal, bronchus, and lung (tbl) and liver cancer burdens among who regions from 1990 to 2019. J Epidemiol Glob Health. (2023) 13:696–724. doi: 10.1007/s44197-023-00144-x 37639192 PMC10686938

[4] Collaborators GNSD. Global, regional, and national burden of disorders affecting the nervous system, 1990-2021: a systematic analysis for the global burden of disease study 2021. Lancet Neurol. (2024) 23:344–81. doi: 10.1016/S1474-4422(24)00038-3 PMC10949203 38493795

[5] Collaborators GF. Burden of disease scenarios for 204 countries and territories, 2022-2050: a forecasting analysis for the global burden of disease study 2021. Lancet. (2024) 403:2204–56. doi: 10.1016/S0140-6736(24)00685-8 PMC11121021 38762325

[6] Steiner TJ Husøy A Stovner LJ. Gbd2021: headache disorders and global lost health - a focus on children, and a view forward. J Headache Pain. (2024) 25:91. doi: 10.1186/s10194-024-01795-2 38831407 PMC11145804

[7] Zhang Y Liu J Han X Jiang H Zhang L Hu J. Long-term trends in the burden of inflammatory bowel disease in China over three decades: a joinpoint regression and age-period-cohort analysis based on gbd 2019. Front Public Health. (2022) 10:994619. doi: 10.3389/fpubh.2022.994619 36159285 PMC9490087

[8] Fan YX Zhang W Li W Ma YJ Zhang HQ. Global, regional, and national impact of air pollution on stroke burden: changing landscape from 1990 to 2021. BMC Public Health. (2024) 24:2786. doi: 10.1186/s12889-024-20230-4 39394088 PMC11470728

[9] Shanshan L Zhihua Z Chengcheng L Huijing C Shangcheng Z. Diabetes in China: burden analysis between 1990 and 2019 and incidence prediction between 2020 and 2030. Chin Gen Practice. (2023) 26:2013–9. doi: 10.12114/j.issn.1007-9572.2023.0009

[10] rdrr.io. Bapc: projection of cancer incidence and mortality data using bayesian apc models fitted with inla(2022). Available online at: https://rdrr.io/rforge/BAPC/ (Accessed December 3, 2024).

[11] Jiyong J Kai H. The motivation, path, and challenges of BRICS countries’ Participation in global health governance. Int Review. (2019), 120–41.

[12] Zou Z Cini K Dong B Ma Y Ma J Burgner DP. Time trends in cardiovascular disease mortality across the brics: an age-period-cohort analysis of key nations with emerging economies using the global burden of disease study 2017. Circulation. (2020) 141:790–9. doi: 10.1161/CIRCULATIONAHA.119.042864 31941371

[13] Campos MR Rodrigues JM Marques AP Faria LV Valerio TS Silva M. Smoking, mortality, access to diagnosis, and treatment of lung cancer in Brazil. Rev Saude Publica. (2024) 58:18. doi: 10.11606/s1518-8787.2024058005704 38747866 PMC11090611

[14] Mathias C Prado GF Mascarenhas E Ugalde PA Zimmer GA Carvalho ES. Lung cancer in Brazil. J Thorac Oncol. (2020) 15:170–5. doi: 10.1016/j.jtho.2019.07.028 32127184

[15] The Climate And Clean Coalition. Brazilian environmental protection policy(2023). Available online at: https://www.ccacoalition.org/partners/Brazil (Accessed December 3, 2024).

[16] Cabral JF Caló R Evangelista FM Reis JB Oliveira J Lima F. Trend analysis of lung cancer incidence and mortality in grande cuiabá, mato grosso, Brazil, 2000 to 2016. Rev Bras Epidemiol. (2022) 25:e220014. doi: 10.1590/1980-549720220014.supl.1 35766771

[17] Smith PH Kasza KA Hyland A Fong GT Borland R Brady K. Gender differences in medication use and cigarette smoking cessation: results from the international tobacco control four country survey. Nicotine Tob Res. (2015) 17:463–72. doi: 10.1093/ntr/ntu212 PMC4402353 25762757

[18] Hao S Wenzhao Z. Higher lung cancer incidence in young women than young men in the United States. J Evidence-Based Med. (2018) 18:216–8. doi: 10.12019/j.issn.1671-5144.2018.04.005

[19] Rongshou Z Ru C Bingfeng H Shaoming W Li L Kexin S. Analysis of the epidemic situation of Malignant tumors in China in 2022. Chin J Oncol. (2024) 46:221–31. doi: 10.3760/cma.j.cn112152-20240119-00035

[20] Bray F Laversanne M Sung H Ferlay J Siegel RL Soerjomataram I. Global cancer statistics 2022: globocan estimates of incidence and mortality worldwide for 36 cancers in 185 countries. CA Cancer J Clin. (2024) 74:229–63. doi: 10.3322/caac.21834 38572751

[21] Hoffman SJ Mammone J Rogers VKS Sritharan L Tran M Al-Khateeb S. Cigarette consumption estimates for 71 countries from 1970 to 2015: systematic collection of comparable data to facilitate quasi-experimental evaluations of national and global tobacco control interventions. BMJ. (2019) 365:l2231. doi: 10.1136/bmj.l2231 31217224 PMC6582269

[22] Yang JJ Yu D Wen W Shu XO Saito E Rahman S. Tobacco smoking and mortality in asia: a pooled meta-analysis. JAMA Netw Open. (2019) 2:e191474. doi: 10.1001/jamanetworkopen.2019.1474 30924901 PMC6450311

[23] Yang D Liu Y Bai C Wang X Powell CA. Epidemiology of lung cancer and lung cancer screening programs in China and the United States. Cancer Lett. (2020) 468:82–7. doi: 10.1016/j.canlet.2019.10.009 31600530

[24] Li C Lei S Ding L Xu Y Wu X Wang H. Global burden and trends of lung cancer incidence and mortality. Chin Med J (Engl). (2023) 136:1583–90. doi: 10.1097/CM9.0000000000002529 PMC10325747 37027426

[25] Wang MY Sung HC Liu JY. Population aging and its impact on human wellbeing in China. Front Public Health. (2022) 10:883566. doi: 10.3389/fpubh.2022.883566 35419339 PMC8995787

[26] National Bureau of Statistics of China. Statistical bulletin on national economic and social development of the people’s republic of China in 2023 (2024). Available online at: https://www.stats.gov.cn/sj/zxfb/202402/t20240228_1947915.html (Accessed December 2, 2024).

[27] Fan Y Jiang Y Li X Li X Li Y Wu H. Burden of lung cancer attributable to occupational carcinogens from 1990 to 2019 and projections until 2044 in China. Cancers (Basel). (2022) 14:3883. doi: 10.3390/cancers14163883 36010878 PMC9405822

[28] Stapelfeld C Dammann C Maser E. Sex-specificity in lung cancer risk. Int J Cancer. (2020) 146:2376–82. doi: 10.1002/ijc.32716 31583690

[29] He B Zhao X Pu Y Sun R Gao X Liu W. Trends and projection of burden on lung cancer and risk factors in China from 1990 to 2060. Thorac Cancer. (2024) 15:1688–704. doi: 10.1111/1759-7714.15332 PMC11293937 38984468

[30] Wu Z Tan F Yang Z Wang F Cao W Qin C. Sex disparity of lung cancer risk in non-smokers: a multicenter population-based prospective study based on China national lung cancer screening program. Chin Med J (Engl). (2022) 135:1331–9. doi: 10.1097/CM9.0000000000002161 PMC9433079 35830209

[31] Wenlin T. Decay and Turmoil: Reflections on the 10th Anniversary of the Arab Spring Vol. 23. Beijing: International Forum (2021) p. 3–17. doi: 10.13549/j.cnki.cn11-3959/d.2021.03.001

[32] Jazieh AR Algwaiz G Errihani H Elghissassi I Mula-Hussain L Bawazir AA. Lung cancer in the middle east and north africa region. J Thorac Oncol. (2019) 14:1884–91. doi: 10.1016/j.jtho.2019.02.016 31668315

[33] Awedew AF Asefa Z Belay WB. National burden and trend of cancer in Ethiopia, 2010-2019: a systemic analysis for global burden of disease study. Sci Rep. (2022) 12:12736. doi: 10.1038/s41598-022-17128-9 35882895 PMC9325704

[34] Gebremariam TH Haisch DA Fernandes H Huluka DK Binegdie AB Woldegeorgis MA. Clinical characteristics and molecular profiles of lung cancer in Ethiopia. JTO Clin Res Rep. (2021) 2:100196. doi: 10.1016/j.jtocrr.2021.100196 34590041 PMC8474241

[35] Mishra S Joseph RA Gupta PC Pezzack B Ram F Sinha DN. Trends in bidi and cigarette smoking in India from 1998 to 2015, by age, gender and education. BMJ Glob Health. (2016) 1:e5. doi: 10.1136/bmjgh-2015-000005 PMC5321300 28588906

[36] Roy MP. Factors associated with mortality from lung cancer in India. Curr Probl Cancer. (2020) 44:100512. doi: 10.1016/j.currproblcancer.2019.100512 31703986

[37] de Bont J Krishna B Stafoggia M Banerjee T Dholakia H Garg A. Ambient air pollution and daily mortality in ten cities of India: a causal modelling study. Lancet Planet Health. (2024) 8:e433–40. doi: 10.1016/S2542-5196(24)00114-1 PMC11774940 38969471

[38] World Health Organization. Current health expenditure (che) as percentage of gross domestic product (gdp) (%)(2024). Available online at: https://apps.who.int/gho/data/node.main.GHEDCHEGDPSHA2011?lang=en (Accessed December 2, 2024).

[39] Mirahmadizadeh A Hassanzadeh J Moradi AM Gheibi Z Heiran A. Projection of the prevalence of tracheal, bronchus, and lung cancer incidence using cigarette smoking prevalence in Iran from 1990 to 2018: a comparison of latent period-based models with standard forecasting models. BMC Public Health. (2024) 24:1896. doi: 10.1186/s12889-024-19407-8 39010019 PMC11251385

[40] Khorrami Z Pourkhosravani M Rezapour M Etemad K Taghavi-Shahri SM Künzli N. Multiple air pollutant exposure and lung cancer in tehran, Iran. Sci Rep. (2021) 11:9239. doi: 10.1038/s41598-021-88643-4 33927268 PMC8085005

[41] Sajadi HS Majdzadeh R. Health system to response to economic sanctions: global evidence and lesson learned from Iran. Global Health. (2022) 18:107. doi: 10.1186/s12992-022-00901-w 36581892 PMC9797877

[42] Salehiniya H Bahadori M Ghanizadeh G Raei M. Epidemiological study of lung cancer in Iran: a systematic review. Iran J Public Health. (2022) 51:306–17. doi: 10.18502/ijph.v51i2.8683 PMC9273489 35866136

[43] Shkolnikov VM Churilova E Jdanov DA Shalnova SA Nilssen O Kudryavtsev A. Time trends in smoking in Russia in the light of recent tobacco control measures: synthesis of evidence from multiple sources. BMC Public Health. (2020) 20:378. doi: 10.1186/s12889-020-08464-4 32293365 PMC7092419

[44] Bai R Dong W Chu M Liu B Li Y. Trends in mortality due to tracheal, bronchial, and lung cancer across the brics: an age-period-cohort analysis based on the global burden of disease study 1990-2019. Chin Med J (Engl). (2024) 137:2860–2867. doi: 10.1097/CM9.0000000000002977 PMC11649273 38311810

[45] Carioli G Malvezzi M Bertuccio P Levi F Boffetta P Negri E. Cancer mortality and predictions for 2018 in selected australasian countries and Russia. Ann Oncol. (2019) 30:132–42. doi: 10.1093/annonc/mdy489 30535287

[46] Schüz J Kovalevskiy E Olsson A Moissonnier M Ostroumova E Ferro G. Cancer mortality in chrysotile miners and millers, Russian federation: main results (asbest chrysotile cohort-study). J Natl Cancer Inst. (2024) 116:866–75. doi: 10.1093/jnci/djad262 PMC11160488 38247448

[47] Jazieh AR Algwaiz G Alshehri SM Alkattan K. Lung cancer in Saudi Arabia. J Thorac Oncol. (2019) 14:957–62. doi: 10.1016/j.jtho.2019.01.023 31122559

[48] Saudi Food and Drug Authority. Saudi food & drug authority standard on plain packaging of tobacco products(2018). Available online at: https://www.tobaccocontrollaws.org/laws/plain-pkg-standard-saudi-arabia (Accessed December 2, 2024).

[49] Zatoński MZ Egbe CO Robertson L Gilmore A. Framing the policy debate over tobacco control legislation and tobacco taxation in South Africa. Tob Control. (2023) 32:450–7. doi: 10.1136/tobaccocontrol-2021-056675 PMC10314007 34824147

[50] Bello B Fadahun O Kielkowski D Nelson G. Trends in lung cancer mortality in South Africa: 1995-2006. BMC Public Health. (2011) 11:209. doi: 10.1186/1471-2458-11-209 21463504 PMC3080816

[51] van Eeden R Tunmer M Geldenhuys A Nayler S Rapoport BL. Lung cancer in South Africa. J Thorac Oncol. (2020) 15:22–8. doi: 10.1016/j.jtho.2019.06.032 31864550

[52] Ayo-Yusuf O Nkosi L Agaku I. E-cigarette use and regulation in South Africa: a synthesis of evidence in response to industry efforts to undermine product regulation. Curr Addict Rep. (2022) 9:363–72. doi: 10.1007/s40429-022-00451-6

[53] Ceicdata. United Arab Emirates Economic Indicators 1950-2024 (2024). Available online at: https://www.ceicdata.com.cn/zh-hans/indicator/united-arab-emirates/population (Accessed September 27, 2024).

[54] Jie C Jieying Z. The problem of marginal people and its governance in GCC countries. Arab World Stud. (2021), 57–75. doi: 10.3969/j.issn.1673-5161.2021.05.004

[55] Al-Zalabani AH. Cancer incidence attributable to tobacco smoking in gcc countries in 2018. Tob Induc Dis. (2020) 18:18. doi: 10.18332/tid/118722 32256282 PMC7107909

[56] Henschke CI Yip R Miettinen OS. Women’s susceptibility to tobacco carcinogens and survival after diagnosis of lung cancer. JAMA. (2006) 296:180–4. doi: 10.1001/jama.296.2.180 16835423

[57] Tse LA Mang OW Yu IT Wu F Au JS Law SC. Cigarette smoking and changing trends of lung cancer incidence by histological subtype among chinese male population. Lung Cancer. (2009) 66:22–7. doi: 10.1016/j.lungcan.2008.12.023 19185950

[58] World Health Organization. Medical services in 90% of the world’s countries are disrupted by the covid-19 (2020). Available online at: https://news.un.org/zh/story/2020/08/1065742 (Accessed December 4, 2024).

[59] Lovlekar SK Wang Y. Impact of the covid-19 pandemic on lung cancer treatment and research. Genes Dis. (2023) 10:1217–9. doi: 10.1016/j.gendis.2023.01.028 PMC10038671 37334159

